# Policy options for strengthening evidence-informed health policy-making in Iran: overall SASHA project findings

**DOI:** 10.1186/s12961-021-00803-0

**Published:** 2022-01-15

**Authors:** Reza Majdzadeh, Haniye Sadat Sajadi, Bahareh Yazdizadeh, Leila Doshmangir, Elham Ehsani-Chimeh, Mahdi Mahdavi, Neda Mehrdad, John Lavis, Sima Nikooee, Farideh Mohtasham, Mahsa Mohseni, Paria Akbari, Mohammad Hossein Asgardoon, Niloofar Rezaei, Narges Neyazi, Saeideh Ghaffarifar, Ali Akbar Haghdoost, Rahim Khodayari-Zarnaq, Ali Mohammad Mosadeghrad, Ata Pourabbasi, Javad Rafinejad, Reza Toyserkanamanesh

**Affiliations:** 1 Community Based Participatory Research Center, Knowledge Utilization Research Center, Tehran University of Medical Sciences, Tehran, Iran; 2 Knowledge Utilization Research Center, University Research and Development Center, Tehran University of Medical Sciences, Tehran, Iran; 3 Knowledge Utilization Research Center, Tehran University of Medical Sciences, Tehran, Iran; 4 Department of Health Policy and Management, School of Management and Medical Informatics, Tabriz University of Medical Sciences, Tabriz, Iran; 5 National Institute of Health Research, Tehran University of Medical Sciences, Tehran, Iran; 6 Nursing Care Research Center, Iran University of Medical Sciences, Tehran, Iran; 7 Endocrinology and Metabolism Research Center, Endocrinology and Metabolism Clinical Sciences Institute, Tehran University of Medical Sciences, Tehran, Iran; 8 McMaster Health Forum and Department of Health Research Methods, Evidence and Impact, McMaster University, Hamilton, Canada; 9 Africa Centre for Evidence, University of Johannesburg, Johannesburg, South Africa; 10 School of Medicine, Tehran University of Medical Sciences, Tehran, Iran; 11 International Campus, School of Public Health, Health Economics and Management Department, Tehran University of Medical Sciences, Tehran, Iran; 12 Trusted Organization for Research and Development, Kabul, Afghanistan; 13 Medical Education Research Center, Health Management and Safety Promotion Research Institute, Tabriz University of Medical Sciences, Tabriz, Iran; 14 HIV/STI Surveillance Research Center and WHO Collaborating Center for HIV Surveillance, Institute for Future Studies in Health, Kerman University of Medical Sciences, Kerman, Iran; 15 School of Public Health, Health Information Management Research Center, Tehran University of Medical Sciences, Tehran, Iran; 16 Department of Medical Entomology and Vector Control, School of Public Health, Tehran University of Medical Sciences, Tehran, Iran; 17 Department of Treatment and Social Support, IRAN Drug Control Headquarter, Tehran, Iran

**Keywords:** Evidence, Policy-making, Knowledge translation, Health research system, Iran

## Abstract

**Background:**

The institutionalization of evidence-informed health policy-making (EIHP) is complex and complicated. It is complex because it has many players and is complicated because its institutionalization will require many changes that will be challenging to make. Like many other issues, strengthening EIHP needs a road map, which should consider challenges and address them through effective, harmonized and contextualized strategies. This study aims to develop a road map for enhancing EIHP in Iran based on steps of planning.

**Methods:**

This study consisted of three phases: (1) identifying barriers to EIHP, (2) recognizing interventions and (3) measuring the use of evidence in Iran's health policy-making. A set of activities was established for conducting these, including foresight, systematic review and policy dialogue, to identify the current and potential barriers for the first phase. For the second phase, an evidence synthesis was performed through a scoping review, by searching the websites of benchmark institutions which had good examples of EIHP practices in order to extract and identify interventions, and through eight policy dialogues and two broad opinion polls to contextualize the list of interventions. Simultaneously, two qualitative-quantitative studies were conducted to design and use a tool for assessing EIHP in the third phase.

**Results:**

We identified 97 barriers to EIHP and categorized them into three groups, including 35 barriers on the “generation of evidence” (push side), 41 on the “use of evidence” (pull side) and 21 on the “interaction between these two” (exchange side). The list of 41 interventions identified through evidence synthesis and eight policy dialogues was reduced to 32 interventions after two expert opinion polling rounds. These interventions were classified into four main strategies for strengthening (1) the education and training system (6 interventions), (2) the incentives programmes (7 interventions), (3) the structure of policy support organizations (4 interventions) and (4) the enabling processes to support EIHP (15 interventions).

**Conclusion:**

The policy options developed in the study provide a comprehensive framework to chart a path for strengthening the country’s EIHP considering both global practices and the context of Iran. It is recommended that operational plans be prepared for road map interventions, and the necessary resources provided for their implementation. The implementation of the road map will require attention to the principles of good governance, with a focus on transparency and accountability.

Video abstract

**Supplementary Information:**

The online version contains supplementary material available at 10.1186/s12961-021-00803-0.

## Background

World leaders assembled at the United Nations on 23 September 2019 to move together to build a healthier world for all by recommitting to achieving universal health coverage (UHC) by 2030, as the core driver of the Sustainable Development Goals (SDGs) [1]. Among affirmations made was the commitment to strengthen the national capacity for health intervention and technology assessment, data collection, and adequate knowledge generation and translation strategies. This recognition echoes a global re-call for evidence-informed health policy- and decision-making. It is widely acknowledged that enhancing the use of evidence in health policy-making is indispensable to improving health system performance and contributing to UHC achievement and the health-related SDGs [2–5].

Many efforts have been made at the national and global levels to link research evidence to policy-making [6–10]. However, obtaining and using high-quality evidence remains challenging, especially in low- and middle-income countries [11–15]. For instance, the Islamic Republic of Iran has introduced some initiatives for promoting evidence-informed health policy-making (EIHP) [16–18]. The most important ones were the establishment of the Center for Strategic Studies (at the Expediency Council), the Social Security Organization Research Institute (at the Social Security Organization), the National Institute of Health Research, the Health Technology Assessment office, the Health Policy Council, the Health Reform Office, the Health System Research Secretariats, the Health Policy-making Council, the Health Transformation Plan Secretariat (at the Ministry of Health and Medical Education [MOHME]), the clinical knowledge management units and the clinical governance units (at medical universities). Concurrent with these developments, the High Council of Health and Food Security, the government's highest health policy authority, announced that it considers documents supported by evidence in their agenda-setting [19].

As a result of these efforts, the use of evidence was considered in developing, implementing and evaluating health policies. However, it is not well institutionalized [20] because of barriers to EIHP in Iran's health system, including the absence of a well-established mechanism for research priority-setting [21], lack of designated individual and organizational support for conducting health policy and systems research, and the lack of a predefined plan for research dissemination and utilization [22]. It seems that the most important barrier is related to weak governance of the health system [20, 23–25], including lack of interest and belief in EIHP and the health research system on the part of policy-makers [26], which are not fully supportive of sustaining EIHP at either the structural or process level. Appropriate interventions for the use of evidence in policy-making should be identified to address these barriers. For this, we need to adopt a systems approach towards health system research for proposing policy options and developing a road map for strengthening EIHP. Any option to improve EIHP needs to be tailored to the health system's characteristics and the country's contextual factors [27].

The main barriers must be identified to establish a road map for improving EIHP. After that, practical and appropriate policy options should be chosen based on global experiences and the specific country context to address identified barriers. Moreover, to ensure progress in achieving EIHP, continuous assessment is advised to monitor the status of EIHP in the country and the impact of proposed interventions.

Despite numerous studies in this field in Iran, their simple compilation did not lead to a specific approach to improving the status quo with respect to EIHP. A holistic approach is desperately needed as a basis for an action plan to enhance EIHP. Hence, a project called SASHA, consisting of three phases, was conducted to develop a road map for strengthening EIHP. Protocols and details published for each phase [28, 29] or their articles are the subject of separate reviews. In the current paper, we present an overview of how these three phases are linked and discuss the results of the project overall. This road map can help institutionalize EIHP in the health system as part of initiatives to adopt the framework for action to improve national capacity for the use of evidence in policy-making [6], and can be adapted by countries with similar challenges and contexts.

## Methods

**Fig. 1:**
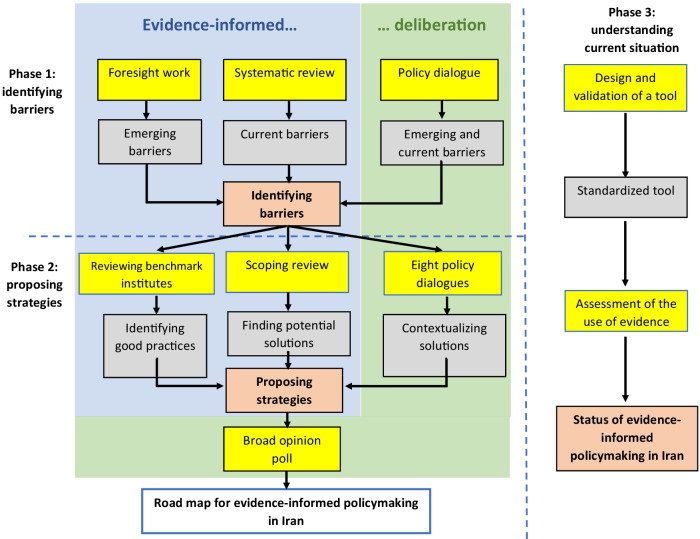
Schematic diagram of the study, including different phases, resulted in a road map of evidence-informed policymaking in Iran†. *Footnote:* † Yellow box: the methodology used; Gray box: Deliverable of each activity; Orang box: output of each phase. Blue and green shaded areas show evidence-informed and deliberative components of the study, respectively

An evidence-informed deliberative process [30] was employed to develop the road map for strengthening EIHP in the Iranian health system. As mentioned, additional details about the methodology of the project were provided elsewhere [28]. In short, the project consisted of three phases, which are illustrated in Fig. 1.

### Phase 1: identifying barriers to EIHP in Iran

This phase was conducted through (1) foresight, (2) systematic review and (3) a policy dialogue. The foresight and systematic review were done to identify and summarize the emerging and current barriers to EIHP in Iran. Then, stakeholder analysis and a policy dialogue were conducted to finalize the barriers to EIHP and root causes. This part of the work was published in a separate article with the full details [29]. In the current paper, we present a piece of that work that summarizes SASHA's overall results.

During foresight, external trends that may affect the future of the health system were extracted through relevant documents. These trends were classified using the STEEP (social, technological, economic, ecological and political) model [31]. Two focus group discussions (FGDs) were held with six relevant experts to discuss and exchange ideas on a subset of trends affecting EIHP, their impact, and the opportunities and threats that affect the future of EIHP in Iran. A list of emerging issues and potential barriers to supporting EIHP in the Iranian health system was the foresight deliverable.

To summarize the factors enabling or hindering the use of evidence in health policy at the macro level in Iran, a search was conducted in PubMed, Scopus, Embase, Health Systems Evidence, and Iranian databases including Magiran, Madlib, Irandoc and the Scientific Information Database [29]. A search for cited references and citation tracking were also carried out in relevant national and international journals, reference lists and related documents. Both English and Persian literature was searched without any time limitation. Two individuals independently conducted screening and data extraction. In the case of disagreement, it was examined by the core research team. Three main categories of knowledge translation (KT) (i.e. push, pull and exchange) were deductively selected. The remaining categories and codes were inductively determined. The deliverable for the systematic review was a list of identified barriers to supporting EIHP in the Iranian health system.

The stakeholders were then identified through a brainstorming session with the entire research team. The stakeholders were categorized based on their role in three KT activities. A policy dialogue was held with 16 stakeholders to get their views regarding barriers and facilitators to EIHP, previously identified through foresight and systematic review. Ten participants worked in push-side organizations, five in pull-side organizations, one in policy support organizations, and four in both push and pull organizations. This session helped inform the stakeholders about the project and finalize the main barriers to supporting EIHP in Iran.

### Phase 2: proposing strategies to address barriers to EIHP

This phase was conducted through (1) scoping review, (2) searching the websites of benchmark institutions, (3) eight policy dialogues and (4) two rounds of opinion polls.

An evidence synthesis was carried out through a scoping review and searching the institutes' websites for good examples of EIHP practices (in push, pull and exchange domains) to identify interventions to address each of the main barriers (eight) found in phase 1. Details of the search method can be found in one of the published articles on identifying interventions [32]. In summary, Scopus, PubMed/Medline and Google Scholar were extensively searched. To systematically retrieve articles on interventions to promote EIHP, relevant keywords in the English language were used. The reference lists of relevant studies were also reviewed. Two individuals independently conducted screening and data extraction to identify studies for interventions to promote EIHP. Disagreement between reviewers was resolved through discussion with the core team of the project. A narrative approach was used to synthesize the findings of the studies.

An overarching review of Iran's documents and the selected institutions' websites was also conducted to extract and compare interventions for supporting EIHP in Iran with those of other countries. The institutions are presented in Additional file 1. The data were compared using a content analysis approach. Based on evidence synthesis, we developed eight policy briefs in which the interventions that had been identified for addressing the barriers were introduced.

Next, eight policy dialogues were held to contextualize the findings. The participants discussed the feasibility of interventions in Iran, key implementation considerations and lessons learned from similar interventions in Iran. The participants of policy dialogue were selected based on respondent-driven sampling. We chose them from different backgrounds and expertise. More details about these policy dialogues are presented in Additional file 2. Data were analysed through manifest content analysis [33]. The contextualized/tailored interventions to address the barriers to EIHP were the deliverable of these dialogues.

Two rounds of Delphi were used to obtain a consensus on the interventions. The experts participating in the process included all who were in previous policy dialogues. A brief about the whole project and the list of interventions were distributed to these 68 experts. They were asked to reflect on whether they agreed or disagreed with each intervention. Interventions were revised given the feedback of 14 experts and were sent to the same experts. The feedback of 17 experts was received during the second round. At least 75% response agreement with each intervention was considered the acceptable level for consensus. Disagreements were discussed in the core research team, and a decision was made to accept or reject based on their justifications. The list of pre-final interventions was sent to relevant individuals and institutions (in push, pull and exchange sides) across the country through official communication by the MOHME. These individuals were asked to indicate whether they agreed or disagreed with interventions within a set period of time (1 month). The final list of interventions was developed given the feedback received from 67 individuals and institutions. We finally organized a FGD with nine experts to develop the road map for strengthening EIHP in Iran. We categorized the interventions into strategies. Then, we drafted the main elements of the road map given the strategies and interventions, including the vision, the policy options and interventions, and the main entity responsible for each of them.

### Phase 3: measuring the use of evidence in health policy-making in Iran

A tool was designed and validated given the policy cycle's main stages, including problem identification, agenda-setting, policy development, implementation and evaluation to assess EIHP status. The SUPPORT approach was used to define the questions to examine the extent of the use of evidence [34]. After standardization of the tool, the status of EIHP was assessed at the macro level of policy-making. To this end, the tool was completed for 14 national health programmes by conducting in-depth interviews with key informants. Data were analysed separately by two individuals using framework analysis.

## Results

### Phase 1: identifying barriers to EIHP in Iran

We identified 97 barriers to EIHP and categorized them into three groups, including 35 barriers on the push side, 41 barriers on the pull side and 21 barriers on the exchange side. In addition to the details of barriers published in a separate paper [29], Table 1 shows the main domains of barriers that were the basis for phase 2 of the project. The lack of or inappropriate enabling process in organizations involving producing and using evidence is one of the main barriers. For instance, the process of allocating research funds to health projects has not been well defined or well institutionalized, or the mechanism of data collection, analysis and sharing is not transparent. We also recognized that despite efforts to establish policy support organizations, several barriers to EIHP referred to the lack of well-structured policy support organizations. Additionally, insufficient abilities and skills and inadequate incentives to motivate researchers to undertake health policy and systems research (HPSR) and to encourage policy-makers to use them were identified as significant barriers to EIHP.

**Table 1:** Main barriers identified to strengthening EIHP

Dimension	Item
Push side	Insufficient skills to conduct HPSR Lack of reward and incentive programmes for researchers to be involved in HPSR Imperfect enabling processes to support producing HPSR
Pull side	Insufficient skills to use HPSR, asking for evidence, and lack of confidence in research for responding to the questions Lack of reward and incentive programmes of decision-makers to use HPSR Imperfect enabling processes to support using HPSR
Exchange side	Lack of well-structured policy support organization(s) Imperfect enabling processes to translate HPSR and prepare it for decision-makers

### Phase 2: proposing strategies to address barriers to EIHP

Table 2 shows the list of 41 interventions identified in phase 2 of the study, which was reduced to 32 interventions after individuals’ and institutions' polling feedback. Twenty-four interventions were recognized through the scoping review and review of benchmark institutions, of which three were excluded from the final list of interventions. Nineteen additional interventions were extracted during policy dialogues, and eight of them were then excluded in the final result.

**Table 2:** Strategies identified to strengthen EIHP

		Decision		Basis for decision	
Strategy	Intervention	Included^a^	Excluded^b^	Review^c^	Expert opinion^d^
Strengthening the education and training system	1. Continuous needs assessment and evaluation of the effectiveness of training courses related to EIHP	√		√	√
	2. Revising the content of curriculums and workshops to increase knowledge and practice of EIHP	√		√	√
	3. Reviewing the method of conducting internships and internships for students, skills development workshops for faculty members and other researchers, and holding study opportunities for faculty members in policy-making organizations	√		√	√
	4. Holding training courses on identifying, evaluating, selecting and applying evidence for health decision-makers, including staff, experts and staff managers of the Ministry of Health and partner organizations	√		√	√
	5. Holding short-term training courses in the field of thinking styles, problem-solving and principles of implementation science for health decision-makers after appointment to managerial jobs by combining practical training methods, mentoring and fellowship	√		√	√
	6. Replacing individual learning with team learning, including researchers and decision-makers as the target group	√			√
Strengthening the incentives programmes	7. Revising the current compulsory criteria and areas of academic promotion with emphasis on measuring the impact of research on health policy, systems and outcomes	√		√	√
	8. Developing appropriate reward and incentive programmes for nonacademic member researchers to persuade them to support EIHP	√			√
	9. Designing metrics to measure research impact on policies or health to evaluate the performance of research institutes and journals	√		√	
	10. Revising the current policies of scientific journals to promote HPSR		√	√	
	11. Revising existing funding mechanisms to support HPSR and KT initiatives	√		√	
	12. Presenting the KT plan when submitting a research proposal as an obligatory prerequisite to all those receiving grants	√		√	
	13. Encouraging and supporting different mechanisms for increasing interactions between policy-makers and researchers	√		√	
	14. Revising some administrative processes, including managers and staff performance evaluation; selection, appointment and change in managers and reward mechanisms to add output-based criteria for EIHP efforts	√		√	√
	15. Establishing an accreditation system for health system managers		√		√
Strengthening policy support organization(s)	16. Capacity-building of research centres and institutes in the field of health policy analysis and evaluation	√			√
	17. Strengthening the multidisciplinary approach to forming research units (such as a research centre or research institute) instead of developing them in fields similar to the academic disciplines	√			√
	18. Division of work and networking between research institutes and higher education in the field of health policy at the national level	√			√
	19. Qualitative assessment of research performance (institutes, universities, centres, etc.)		√		√
	20. Strengthening the role of exchange organizations through reviewing the mission and responsibilities, designing and implementing merit selection and a meritocracy system for managers and employees, active participation of stakeholders in the composition of exchange organization governance bodies, and using existing capacities within and outside the organization of policy-making organizations to analyse and evaluate health policies	√		√	√
	21. Establishment of health policy analysis units in policy-making organizations		√		√
Strengthening the enabling processes	22. Make transparent details of the decision-making process about funding research projects	√		√	√
	23. Prepare, approve and communicate guidelines/protocols for conflict of interest	√		√	√
	24. Optimize conducting HPSR by setting research priorities and defining research questions based on the needs and active participation of all stakeholders (including the public)	√		√	√
	25. Strengthen the active participation of stakeholders (including the public) in HPSR	√		√	√
	26. Improving the quality of HPSR	√		√	√
	27. Requiring the registration of research activities in the national system and anticipating the processes to prevent parallel research activities	√			√
	28. Needs assessment of the required number of researchers active in HPSR and reviewing the method of attracting and retaining these researchers	√			√
	29. Obligation to attract research funding from policy-making organizations to solve real health problems in exchange for a share of researchers' salaries		√	√	√
	30. Preparation and implementation of evidence-aware policy-making protocol at all stages with an emphasis on transparency and accountability	√			√
	31. Using the criterion of “evidence-based” in prioritizing and allocating health resources	√		√	√
	32. Ensuring, empowering and having a transparent process of stakeholder participation (including people) in health policies		√		√
	33. Review the method of selection and appointment of managers and experts in policy-making organizations by adding the criteria of having the knowledge and skills needed for evidence-based decision-making	√			√
	34. Prepare, approve and communicate guidelines/protocols for conflict of interest management for health system decision-makers and policy-makers		√	√	√
	35. Establish a comprehensive system of monitoring and evaluation	√		√	√
	36. Clarification of information on full ordering details; the appointment of an organization/researcher producing evidence and contracts		√		√
	37. Develop and implement instructions for the process of ordering and concluding research contracts, monitoring and data exchange		√		√
	38. Modify the health information system so that the type of data collected is appropriate for the needs of policy-makers, has acceptable quality and transparency in the process of ownership, production and sharing of data, complies with confidentiality principles, and avoidance of duplicate data collection is guaranteed	√		√	√
	39. Integration of decision-making units (at the stage of proposing new structures and formulating processes) within the policy-making organization	√			√
	40. Improve the interaction of ministries and organizations regarding health sector interventions (e.g. in the High Council for Health and Food Security) to clarify responsibilities, require all organizations to provide evidence for programmes, develop a joint action plan and evaluate the performance of each outcome-based stakeholder	√			√
	41. Providing funds/grants to produce evidence in the long-term health planning of the country	√		√	

^a^Interventions included in the final road map.

^b^Interventions proposed during the review of evidence or policy dialogue that were not included after two rounds of broad opinion polls and were excluded from the final road map.

^c^Interventions listed from the review of the literature or those among practices of benchmark institutions.

^d^Interventions proposed during policy dialogues.

The final list was classified into four strategies: strengthening the education and training system (6 interventions), the incentive programmes (7 interventions) and the structure of policy support organizations (4 interventions), and enabling processes to support EIHP (15 interventions).

### Phase 3: measuring the use of evidence in health policy-making in Iran

By using the tools designed in this study, the status of the use of evidence in health policy-making in Iran was examined in two parts: (i) programme development (including identifying and putting the problem on the agenda, recognizing possible solutions, selecting appropriate solutions and reviewing implementation considerations), and (ii) after the implementation of the programme (Table 3).

**Table 3:** The status of use of evidence in health policy-making in Iran

Identifying the problem	Putting on the agenda: In two of 14 programmes, the problem is raised through the media (as a reflection of the demands of the people) Reviewing the causes: The causes of the problem were investigated in one third of the programmes by using a specific study
Recognizing solutions	A range of actions were taken from 14 programmes: - Preliminary study to evaluate effectiveness, economic evaluation, searching resources, and application of WHO recommendations and expert opinion: 1 - Study of economic evaluation and expert opinion: 1 - Search for resources and application of WHO recommendations: 1 - Application of WHO recommendations: 5 - Search for resources and opinions of experts: 3
Contextualizing solutions	Identify the required change scope: In one-third of the cases, a specific study was conducted for adaptation to local conditions. In one-third of the programmes, either the opinion of experts or policy-makers' previous experiences was used Identify the limits of change: Took place in 1/12th of the programmes to identify the permissible limits of change for localization Budget impact estimation: About one-sixth of the cost estimates were performed at the time of programme design Pilot and modification based on it: Done in one-fifth of the programmes Stakeholders' opinion: Half of the programmes used the opinions of stakeholders (except programme designers)
Implementation solutions	The study of the obstacles and facilitators of implementing the solution was done in four of the 14 programmes at the time of design and in two of the 14 programmes at the time of programme implementation
None of the programmes at the time of design had prepared a comprehensive protocol for monitoring and evaluation. In nine of the 14 programmes, this was done after the programme's implementation	Monitoring implementation and evaluating impacts

(i) Use of evidence during programme development: When developing policies, during the stage of identifying and putting the problems on the agenda, the policy-maker's experience has a leading role, prioritization is weak, and the function of civil society and the nongovernmental sector is minimal. In order to choose the appropriate solution to deal with health problems, one must first identify the causes of the problem and the causes of its persistence. Only about one third of the programmes examined the causes of the problem before selecting the solutions. WHO’s direct recommendations were the basis of half of the policies. In general, systematic review and the use of secondary data to identify solutions were forsaken. Also, before implementing the solution and revisions based on the pilot study, economic evaluations were done in a small number of programmes. Contextualization was not considered in 30% of programmes. The development of a monitoring and evaluation plan was neglected at the time of policy development.

(ii) Use of evidence after implementing programmes: It was found that some programmes, although they did not make extensive use of evidence in their development phase, have taken steps to improve the programme after its national scale-up. As shown in Table 3, a small number of programmes had been monitored and evaluated systematically through a defined project.

## Discussion

We developed a national road map for strengthening EIHP by providing interventions to address the barriers to institutionalizing EIHP in Iran. We also sought to assess the current status of the use of evidence in health policy-making.

The assessment of EIHP showed that the use of evidence in each stage of the policy cycle is not optimal. The root causes of the problems are not examined systematically; problem clarification, which is the first step for selecting appropriate solutions, is overlooked. When choosing and implementing solutions, the most crucial steps, including using policy-makers and stakeholders' views and experiences, synthesis of evidence, economic evaluations and pilot study, are not adequately considered. The policy-makers and managers do not take advantage of using evidence during the implementation and evaluation of interventions. The findings also showed that the WHO recommendations play a significant role in selecting and implementing solutions. Because of the substantial power of policy-makers and managers in shaping policies, criteria for choosing them, professional development and improving their competencies in evidence-informed decision-making must be considered priority actions.

Even though phase 3 of the study was carried out simultaneously with the other two phases, it was impossible to use its results to identify the barriers. Still, in addition to our intention to obtain data on the current status of EIHP, this can be triangulated with the barriers identified in phase 1. The results of phase 3 showed that the EIHP is not well used in the policy-making and implementation process, and phase 1 showed that this is due to the lack of necessary skills among policy-makers and researchers. Researchers can be promoted based only on the number of articles published, and making health system changes is not adequately valued in their promotion criteria. Policy-makers are not encouraged to use evidence, either by considering the support of evidence as a criterion in accepting a programme or by including the use of evidence in their performance assessment.

The relation between phases 1 and 2 of the study is also well established. The weaknesses of researchers' and policy makers' abilities and skills identified as barriers in phase 1 are covered in the first strategy and subsequent interventions in phase 2 (Table 2, numbers 1 to 6, under the category “strengthening education and training system”). The issues raised as inadequate incentives are addressed in the second set of strategic interventions (numbers 7 to 15), strengthening the incentive programmes. The lack of well-structured policy support organizations created the third strategy and related interventions (16 to 21). Lastly, the barriers to the processes were addressed in interventions 22 to 41 under the “strengthening enabling processes” strategy.

### Strengths and weaknesses

This study's strength is its methodology in reviewing the evidence and best practices to tailor an appropriate solution for strengthening EIHP through a deliberative approach with stakeholder participation. The majority of national-level road maps are created using expert views. However, this study sought to walk through the logical evolution of identifying challenges and then developing interventions. As a result, the proposed road map is specifically based on Iran's present problems. Another advantage of this study is that it reviewed successful international organizations and their experiences, which are not necessarily present in peer-reviewed publications. Moreover, numerous rounds of discussions and reviews were held to adopt the interventions for Iran, review their strengths and weaknesses, and employ a systemic view of their effects.

The main weakness of this study is the scarcity of evidence in this area. As mentioned earlier, there is very little research evidence available, and it cannot be argued that a high level of evidence supports the recommendations. Many experiences in Iran have overlooked assessing the effect of interventions, and so the research team had to use implementation evidence from other countries, if available. Of course, there are limitations in generalizing the results from other countries to Iran. The views of experts and stakeholders were used to overcome this issue. Although this was the only possible solution, it is not necessarily correct. Finally, while the road map was developed based on the Iranian context, it can be a good source of information for other countries facing similar challenges.

### Findings in relation to other studies

The barriers to EIHP identified in our study differ somewhat from those reported by other scholars, showing that although some of them are common in all countries, some are context-specific [13, 14, 35]. Since the integration of medical education into health service delivery in Iran, both systems have been governed by the MOHME [36]. Despite the platform provided by this integration of education and research with health services [36, 37], this mutual platform is not appropriately used. It shows that the research funding bodies can play an essential role in enhancing the EIHP in a situation like Iran [38]. Moreover, most of these challenges are wicked problems and have a complex causal network. It is inevitable that a systems approach is needed for dealing with them [39].

### Implications for policy and practice

The findings in Table 2 also proposed a number of interventions, categorized into strengthening education and training systems, incentive programmes, policy support organizations, and enabling processes to support EIHP. The intervention for strengthening education and training systems ranges from needs assessment to increasing skills at various professional stages. Given the specialization of roles and the significance of collective synergy in decision-making, capacity-building should be team-oriented. Both evidence producers and users should have the necessary skills to play their collaborative roles in a team. The interventions of the incentive programmes showed the need to shift from merely encouraging article publication to evidence utilization. The interventions to support policy support organizations emphasize optimal utilization of existing research centres' capacities, coordination and division of tasks among organizations, and offering roles in exchange organizations to reinforce ties between evidence producers and users. The interventions to strengthen enabling processes, which have the highest number of interventions proposed in the road map, mainly focus on transparency, accountability and stakeholder participation in both sides of health research and health systems. Further progress in the use of evidence requires fundamental modifications to institutionalize good governance in the health research system and the health system [20, 24, 25].

To implement the identified interventions, we need to start from governance, as suggested in the previous studies [25]. The interventions required to reform governance depend on the structure of the health system and its ties with the health research system, whether they have separate governance or joint governance. In this regard, the role of funding organizations is becoming more prominent and fundamental [40]. The governance of the health research system by the MOHME has advantages and disadvantages [5]. Although in an integrated structure, coordination between various sections of the MOHME with the research authorities is structurally easier [41], without coordinated processes this advantage cannot not be realized. The procedures should be regulated so that cooperation and accountability between different administrative bodies are clearly defined. As proposed by previous studies, establishing mechanisms for more transparency and accountability, and public engagement is essential to improving the governance arrangements and performing more effectively [20, 24].

It is worth noting that our study was conducted before the COVID-19 pandemic. The world and Iran amid COVID-19 have experienced new conditions and learned important lessons that must be considered in health research systems and in defining interventions. First, we understand that countries and health systems were fragile in response to the COVID-19 pandemic. They could not withstand the shock caused by the pandemic. Second, the health research systems became accountable more than ever for timely evidence to deal with the pandemic. Never have society and decision-makers been so thirsty for research evidence [42]. Examples of questions that research systems needed to urgently address were related to vaccines, medicines effective for treating COVID-19 and effective preventive measures. The experience of COVID-19 suggests that an important consideration in designing research systems is their responsiveness in times of crisis. We need a resilient research system that can be accountable during crises [43]. These should be addressed in addition to the suggested interventions in the road map. It appears that the health research system should be considered as a common good for health [44], and investment in a resilient health research system must be viewed as step zero of building an accountable system [45]. This way of thinking is new to health research system design and emerged after dealing with the COVID-19 pandemic.

The destiny of the proposed road map would not be different from the 14 health programmes reviewed in phase 3 as study subjects. The authors of this article have made every effort to use local and global evidence to find the best solutions to strengthen EIHP in Iran and to aggregate them systematically. But the details of the implementation plan for the road map are crucial and are needed. An action plan and resources are required for each of the 32 interventions listed in Table 2. Strong governance is a prerequisite; transparency is necessary, and the road map and its executives must be accountable. As the first step, the road map needs to be legitimized, approved and endorsed by the MOHME and other stakeholders.

### Implications for future research

It is worth noting that about 65% of these interventions proposed in the road map were obtained based on the scoping review and best practices worldwide. This highlights the current scant evidence on effective interventions to strengthen EIHP. The effectiveness of these interventions was judged mainly based on stakeholder views or from institutions reviewed as having successful examples. Therefore, what is recommended in this study and is available globally for enhancing EIHP suffers from a lack of rigorous evidence, revealing the current knowledge gap about the effectiveness of interventions for improving EIHP.

## Conclusion

Various initiatives are being launched at the global and regional levels to enhance EIHP. But to implement them in each country, a road map must be developed, given the context and resources. The road map developed in this study provides a practical framework for charting the country’s path towards strengthening EIHP, taking into account the global recommendations and the context. Therefore, it is recommended that an operational plan be prepared for each intervention included in the road map, and then the resources required for its implementation should be estimated. However, the road map must first be approved as a comprehensive plan for enhancing EIHP and then communicated to the related organizational sectors (e.g. MOHME) for implementation.

## Supplementary Information

**Additional file 1.** The list of selected institutions have been reviewed.

**Additional file 2.** Details of policy dialogues have been conducted to contextualize the intervention.

## Data Availability

All data generated or analysed during this study are included in this published article and its additional files.

## Abbreviations

EIHP: Evidence-informed health policy-making
FGD: Focus group discussion
MOHME: Ministry of Health and Medical Education
STEEP: Social, technological, economic, ecological and political
SDGs: Sustainable Development Goals
UHC: Universal health coverage
