# Supplementary material for: Policy options for strengthening evidence-informed health policy-making in Iran: overall SASHA project findings
Source: Health Res Policy Syst. 2022 Jan 15;20:10. doi: 10.1186/s12961-021-00803-0 (PMC8760808; doi:10.1186/s12961-021-00803-0)
Supplement: Supplementary file 2 — Additional file 1. The list of selected institutions have been reviewed. [file 12961_2021_803_MOESM2_ESM.docx]

**Additional file 1: The list of selected institutions to review**

Pushing organizations:

1. Qeen Mary University of London
2. Mc Master University
3. Moash University
4. Ebonyi State University Abakaliki Nigeria

Pulling organizations:

1. Australia ministry of health
2. Canada department of health
3. UK health department

Exchange organizations:

1. Behavior Works Australia
2. Cochrane Australia
3. Sax Institute Australia
4. Mc Master health forum
5. Policy institute in KCL(UK)
6. Knowledge to Policy Centre (Lebanon)
7. EPPI Centre(UK)
8. Africa Centre for Evidence
9. African Institute for Health Policy & Health Systems, Reviews and Knowledge Translation (South Africa)
