# Supplementary material for: Policy options for strengthening evidence-informed health policy-making in Iran: overall SASHA project findings
Source: Health Res Policy Syst. 2022 Jan 15;20:10. doi: 10.1186/s12961-021-00803-0 (PMC8760808; doi:10.1186/s12961-021-00803-0)
Supplement: Supplementary file 3 — Additional file 2. Details of policy dialogues have been conducted to contextualize the intervention. [file 12961_2021_803_MOESM3_ESM.docx]

**Supplementary file 2: Details of policy dialogues conducted to contextualize the interventions**

| Agenda | Time | Durations (min) | Rate of Participation (%) |
| --- | --- | --- | --- |
| Discuss interventions identifies to improve incentive programs of health researches | July 7, 2019 | 127 | 71 |
| Discuss interventions identifies to strengthen enabling process of pushing organizations | July 8, 2019 | 116 | 50 |
| Discuss interventions identifies to strengthen enabling process of pulling organizations | July 10, 2019 | 98 | 31 |
| Discuss interventions identifies to strengthen enabling process of exchange organizations | July 14, 2019 | 126 | 50 |
| Discuss interventions identifies to improve incentive programs of policymakers | July 21, 2019 | 121 | 67 |
| Discuss interventions identifies of empowering the health researches | July 22, 2019 | 105 | 51 |
| Discuss interventions identifies to strengthen structures of policy support organizations | July 24, 2019 | 194 | 71 |
| Discuss interventions identifies of empowering the policymakers | Augest 4, 2019 | 118 | 57 |
